# Phylogenetic Diversity of Plant and Insect Communities on Islands

**DOI:** 10.1002/ece3.70660

**Published:** 2024-12-02

**Authors:** Thomas Leclère, Pille Gerhold

**Affiliations:** ^1^ Institute of Agricultural and Environmental Sciences Estonian University of Life Sciences Tartu Estonia

**Keywords:** community ecology, island biogeography, phylogenetic diversity, plant–insect interactions

## Abstract

Interactions between plants and insects have long fascinated scientists. While some plants rely on insects for pollination and seed dispersal, insects rely on plants for food or as a habitat. Despite extensive research investigating pair‐wise species interactions, few studies have characterized plant and insect communities simultaneously, making it unclear if diverse plant communities are generally associated with diverse insect communities. This work aims to better understand the historical and evolutionary relationships between plant and insect phylogenetic diversity (PD) on islands. We hypothesized that phylogenetically diverse plant communities (i.e., high PD) support diverse insect communities, with the relationship varying with island isolation, area, age, and latitude. Species lists for plants and insects were compiled from the published literature, and plant PD was calculated using ´standardized mean pairwise distance´ (SES.MPD) and ´standardized mean nearest taxon distance´ (SES.MNTD). For insects, PD was estimated using the number of genera, families, and orders. We found that plant diversity in evolutionary recent times (SES.MNTD) is associated with recent insect diversity (number of genera), but no relationship was found between plant and insect diversity across whole phylogenies (plant SES.MPD vs. number of insect families). Distant islands generally support high PD of plants (high SES.MPD and SES.MNTD) and insects (low number of genera). Plant and insect PD was generally high on small islands, except for plant SES.MPD revealing no relationship with island size. Insect PD was somewhat higher on young islands (low number of families), whereas there was no relationship between island age and plant PD. Plant SES.MPD was higher on high latitude islands, yet we did not find significant relationships between the latitude and the metrics of insect PD or plant SES.MNTD. These findings suggest that protecting high plant PD may also help conserve high insect PD, with a focus on small and distant islands as potential hotspots of phylogenetic diversity across multiple taxa.

## Graphical abstract

High plant diversity in evolutionary more recent times is significantly associated with high recent insect diversity.

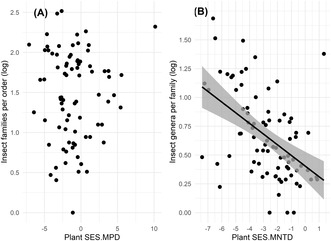

## 1 Introduction

A major goal of biological research is to document patterns of biodiversity, an essential step in order to conserve it in the face of global change threats (Jaureguiberry et al. 2022). Biodiversity is unevenly distributed across the globe, and there is increasing urgency to prioritize the conservation of biodiversity hotspots (Myers et al. 2000). Islands are biodiversity hotspots due to high rates of species endemism, containing about 17% of the world's plant diversity (Kier et al. 2009). Unfortunately, islands also account for the highest numbers of species endangerment and extinction (Fernández‐Palacios et al. 2021), highlighting the severity of global threats to this unique biodiversity. Islands are also ideal for studying processes that shape local communities due to their isolation and well‐defined boundaries.

The high biodiversity on islands can be partly explained by the island's physical parameters such as the distance to the mainland, island area, and age, as they define the dynamics between colonization and extinction rates, as postulated by the theory of island biogeography (MacArthur and Wilson 2001; Weigelt et al. 2015). This equilibrium between colonization and extinction has been revisited multiple times since its first publication (Losos, Ricklefs, and MacArthur 2010), and there is an ongoing interest in studying speciation and endemism (Emerson and Gillespie 2008; Parent, Caccone, and Petren 2008).

While patterns of biodiversity have been extensively studied for plants (Qian, Ricklefs, and White 2004; Kreft and Jetz 2007) and other groups of organisms [e.g., birds (Jetz et al. 2012; Voskamp et al. 2017)], multitrophic approaches are not so common (but see Schuldt et al. 2018, 2019; Seibold et al. 2018), such as the relationship between insects and plants. However, plant‐insect interactions, both trophic and non‐trophic (Kawatsu et al. 2021), are key components of natural communities (Van Der Plas, Anderson, and Olff 2012; Albert et al. 2022). Moreover, biotic interactions can have evolutionary impacts on species´ diversity patterns via increasing diversification rates in positive interactions such as insect pollination for plants, or decreasing diversification in negative interactions such as predation or competition (Zeng and Wiens 2021). Due to the lack of multitrophic studies, it remains unclear whether plant and insect diversity are linked or the circumstances in which they may not be linked (Lewinsohn and Roslin 2008; Kemp, Linder, and Ellis 2017). Previous studies have emphasized strong relationships between plants and insects (Murdoch, Evans, and Peterson 1972), and it has even been suggested that insect diversity can be predicted from plant diversity (Zhang et al. 2016). While plant diversity has been shown to enhance insect diversity in multiple cases (Nicholls and Altieri 2013; Mata et al. 2021), the relationship varies across ecosystems and taxonomic groups (Schulze et al. 2004) and across habitat parameters such as area and isolation (Aizen et al. 2016).

Classic studies of biodiversity are based on species richness (i.e., the number of species), yet for understanding the underlying ecological and evolutionary mechanisms, biodiversity should be viewed as the diversity of evolutionary lineages that have accumulated and dispersed in time and space, i.e. phylogenetic diversity (PD; Donoghue 2008). Phylogenetic studies have become increasingly common due to the widespread use of molecular methods (Amit Roy 2014), and phylogenetic trees have become more accurate and robust (Reaz, Bayzid, and Rahman 2014). This improved understanding of phylogenetics allows for a better understanding of the ecological and evolutionary processes that shape biological communities (Cavender‐Bares et al. 2009; Gerhold et al. 2015) as well as the consequence of the community interaction on the community dynamic (Cavender‐Bares et al. 2009).

Phylogenetic diversity is high in a community that consists of far‐related species and low in a community of closely related species (Webb 2000; Webb et al. 2002). Phylogenetic relatedness between co‐existing species can be measured with indices such as the ‘standardized mean pairwise distance’ (SES.MPD) and ‘standardized mean nearest taxon distance’ (SES.MNTD). While the first index measures phylogenetic distances across the whole phylogenetic tree by averaging all species pairwise distances (i.e., it incorporates both ancient and recent diversifications), the latter index measures phylogenetic distances among the tips of the phylogeny, capturing only recent diversifications. The standardized indices are independent of species richness because they account for richness‐related biases by assessing whether a given number of species in a community are more or less phylogenetically related than expected by chance (Miller, Farine, and Trisos 2017).

Finally, the phylogenetic structure of present‐day communities can be the result of both recent community assembly and deep‐past processes (Götzenberger et al. 2012; Gerhold et al. 2018). Such processes can be split into macro‐evolutionary processes (e.g., diversification rate, cospeciation), environmental influence (e.g., habitat size, isolation), and trophic interactions (e.g., presence of host communities) (Gerhold et al. 2018). Distinguishing deep‐past processes from community assembly is hard to make due to the multiple causes of phylogenetic community structure (Cavender‐Bares et al. 2009) and it remains a major debate between ecologists.

In this work, we aim to better understand the evolutionary history of island communities by investigating the relationship between plant and insect phylogenetic diversity with a focus on islands with a widespread global distribution. Because it is well established that island diversity is strongly influenced by island isolation, area, and age (Badano et al. 2005; Fattorini 2011; Barajas‐Barbosa et al. 2020), it is predicted that these factors will modulate the insect‐plant diversity relationships. Moreover, given the well described latitudinal gradient in diversity (Hillebrand 2004; Mittelbach et al. 2007), it is further predicted that insect‐plant diversity will be higher at low latitudes than at high latitudes.

We first hypothesize that, given the long evolutionary history of several highly specific plant‐insect interactions (Bronstein, Alarcón, and Geber 2006; Labandeira 2013), plant communities of high PD are generally associated with insect communities of high PD. We further hypothesize that isolated islands harbor communities of low PD due to in situ radiation, occurring more frequently on remote islands, especially for plant and insect species (Losos and Ricklefs 2009; but see Cerca et al. 2023). Alternatively, isolated islands may have been colonized by distantly related lineages leading to high PD (Cavender‐Bares et al. 2009). Large islands may support high PD due to the higher variability of environmental conditions, especially when there are elevational gradients (Weigelt, Jetz, and Kreft 2013) or low PD due to the big area available for in situ radiation. On old islands, PD may be high because there may have been more time for the accumulation of distantly related lineages which increases PD or PD may be low due to the predominance of in situ radiations.

## 2 Material and Methods

The data were collected using the databases Google Scholar (for papers published from 1945) and JSTOR (for papers published between 1700 and 1945). We considered all papers containing species lists of plants and insects collected in the same locations. We used the following search terms:

“Species list” OR “Species richness” OR “Species diversity” OR “Inventory” OR “Checklist” OR “Survey” AND ((“Plant diversity” OR “Plant survey” OR “Plant community”) AND (“Insect diversity” OR “Insect survey” OR “Insect community”)).

Papers focusing on pest insects and/or agricultural studies were excluded as they are not representative of the natural environment. A total of 81 pairs of plant‐insect species lists were compiled from 53 publications and book chapters (Appendix S2). The papers contained full species lists, with no native and non‐native species separated, hence it was not possible to separate native and non‐native species in the current study. We excluded one location (Rose Atoll) with both plant and insect species richness < 5.

The final dataset represented a diverse range of islands, both oceanic (*n* = 55) and continental (*n* = 26), with a wide span of island parameters such as distance from mainland, area and age. We measured the island's minimal distance to the mainland using Google Maps, whereas the area was determined using Global Island Explorer (Sayre et al. 2019). The estimated age of the islands was determined by the information in the published literature (available in Appendix S1 and S2).

Care was taken to here only include studies that sampled above‐ground insects, with no studies involving below‐ground insects. The methods collecting above‐ground insects were comparable across the studies, including Malaise traps (e.g., Early 1995), sweep nets (Hwang et al. 2022; Meads and Fitzgerald 2001), hand searching (Meads and Fitzgerald 2001; Early 1995), black light traps (Lim et al. 2013; Ryu et al. 2021), pitfall traps (e.g., Ryu et al. 2021), or yellow pan traps (e.g., Early 1995).

Plant species names were updated and standardized using The Plant List (2013) using “GBOTB.extended.TPL” in the package “U.Taxonstand” (Zhang and Qian 2022). We constructed a phylogeny of plant species based on the megaphylogeny for seed plants (Smith and Brown 2018, updated by Jin and Qian 2019), using the package “V.PhyloMaker2” (Jin and Qian 2022).

We used the package “picante” to calculate the indices of plant phylogenetic diversity: ‘mean pairwise distance’ (MPD) and ‘mean nearest taxon distance’ (MNTD) across the plant species in each community (Webb 2000; Webb et al. 2002; Pavoine and Bonsall 2011). MPD measures PD across the whole phylogeny (including both ancient and recent diversification), whereas MNTD measures PD among the tips of the phylogeny (including only recent diversifications).

To make the indices independent of species richness, we used the standardized effect sizes of both indices (Miller, Farine, and Trisos 2017):

$$\mathrm{SES}.\mathrm{MPD}=\left(\text{MPDobs}-\text{MPDnull}\right)/\mathrm{sd}\left(\text{MPDnull}\right)$$

$$\mathrm{SES}.\text{MNTD}=\left(\text{MNTDobs}-\text{MNTDnull}\right)/\mathrm{sd}\left(\text{MNTDnull}\right)$$

where MPDobs/MNTDobs is the observed value in a community, MPDnull/MNTDnull is the average of the expected value in the randomized communities (*n* = 1000 randomizations), and sd(MPDnull)/sd(MNTDnull) is the standard deviation of 1000 null values. Negative values of SES.MPD and SES.MNTD indicate that species tend to be more closely related in a community, whereas values greater than zero indicate that species tend to be more distantly related.

Due to the lack of a global phylogeny for insect species, we were not able to calculate the indices of phylogenetic diversity for insects. We, therefore, used the number of taxonomic groups as a proxy for phylogenetic diversity in insects (Zou et al. 2020). We calculated the average number of insect families per order as a proxy for more ancient diversifications (similar to plant SES.MPD), and the average number of insect genera per family as a proxy for more recent diversifications (similar to plant SES.MNTD). Though simple and coarse, the numbers of families and genera have been used as proxies for evolutionary relationships when complete phylogenies are not available (e.g., Keith et al. 2005; Brehm, Strutzenberger, and Fiedler 2013). A low value of these proxies indicates a phylogenetically diverse community (i.e., high PD) whereas a high value indicates a phylogenetically poor community (i.e., low PD).

Positively skewed data was log‐transformed (insect diversity indices, island area, island age) or square root‐transformed (distance from mainland) to achieve a normal distribution. We performed Moran's *I* test on the plant and insect diversity metrics to detect spatial autocorrelation. We studied the relationships between plant and insect phylogenetic diversity (plant SES.MPD vs. number of insect genera per family, and plant SES.MNTD vs. number of insect families per order) across all islands, using a spatial autoregressive model, and calculating correlations between the spatially detrended variables. We studied the effect of island parameters (distance, area, age, and type) on plant and insect phylogenetic diversity by using a linear regression model with generalized least squares (GLS) that accounts for spatial autocorrelation. We chose the most suitable model based on the Akaike Information Criteria (AIC). All data analysis was conducted in R Studio (R Core Team 2022).

## 3 Results

**FIGURE 1:**
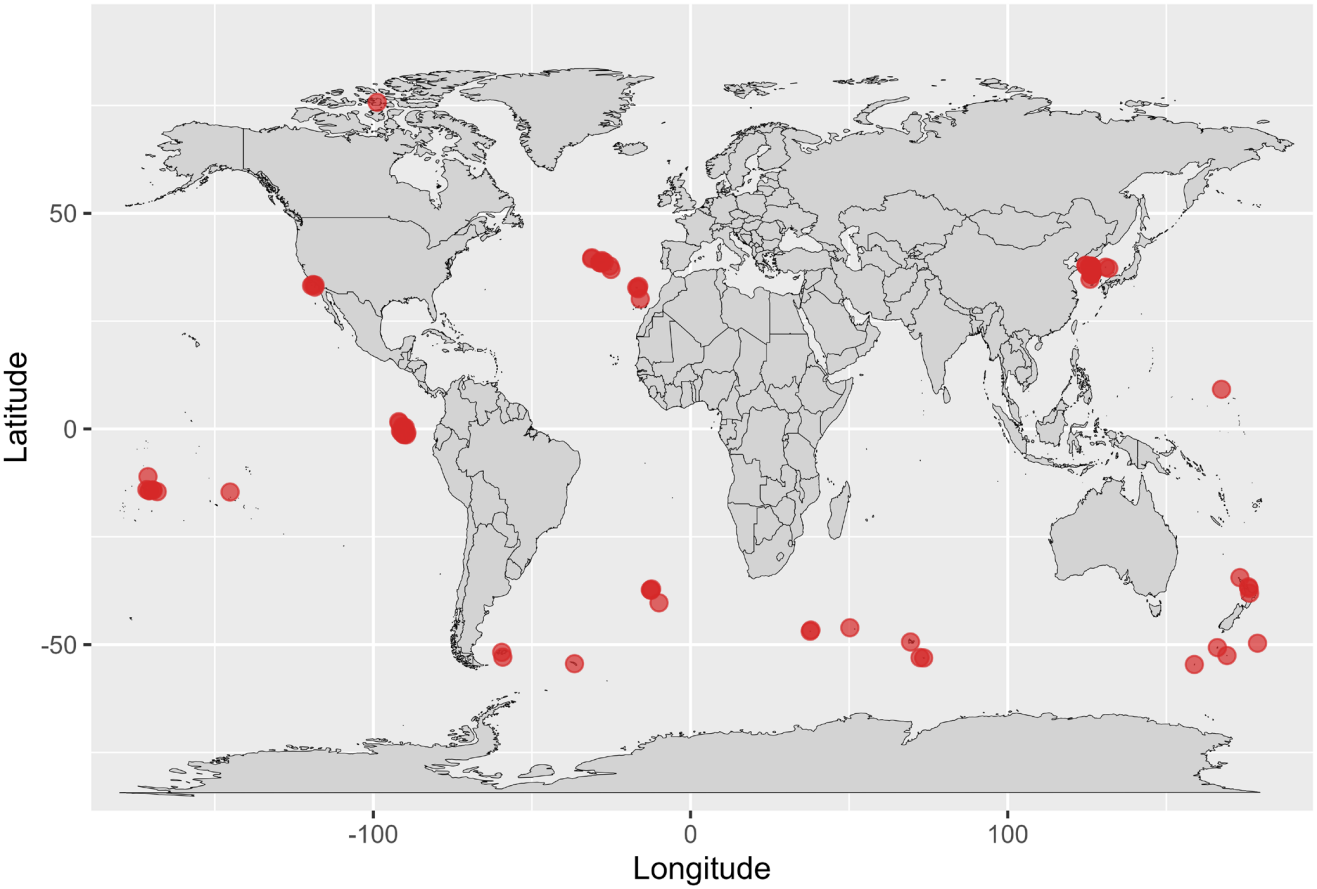
Location of the studied islands (*n* = 81; red dots).

In total, 81 islands were included in the analyses, dispersed along a wide latitudinal gradient from 75.72° N on Bathurst Island in Canada to 54.62° S on Macquarie Island in Australia (Figure 1 and Appendix S2). The islands also represent a wide range of size (0.147 km^2^ for Dokdo to 12,173 km^2^ for Falklands), age (from 0.1 Ma for Aunu'u to 910 Ma for Maldo and Myeongdo), and distance from mainland (0.36 km for Ganghwado to 4739.54 km for Takapoto).

Plant SES.MPD and SES.MNTD had weak to moderate spatial autocorrelation (Moran *I* = 0.41, and 0.26, respectively), and the indices of insect PD had weak spatial autocorrelation (Moran *I* = 0.23 for families per order, and 0.23 for genera per family). Accounting for spatial autocorrelation, plant SES.MNTD was most explained by island parameters (*R* ^2^ = 0.3423), whereas plant SES.MPD was the least explained by island parameters (*R* ^2^ = 0.2568) (Table 1).

**TABLE 1:** GLS models for the effects of island parameters (distance from mainland, area, age, and latitude) on plant and insect diversity metrics (plant SES.MPD, plant SES.MNTD, number of insect families per order, number of insect genera per family), taking into account spatial autocorrelation. Significant variables for each model are in bold. *R* ^2^ values are displayed for each model. Df = 74.

	Plants				Insects			
	SES.MPD		SES.MNTD		Families/Order (log)		Genera/Family (log)	
	*t*	*p*	*t*	*p*	*t*	*p*	*t*	*p*
(Intercept)	**−3.87**	**< 0.001**	**−6.15**	**< 0.001**	**6.23**	**< 0.001**	**4.16**	**< 0.001**
Distance (sqrt)	**3.02**	**0.003**	**4.29**	**< 0.001**	−1.53	0.128	−1.85	0.067
Area (log)	−0.51	0.609	**−4.75**	**< 0.001**	**6.67**	**< 0.001**	**5.11**	**< 0.001**
Age (log)	−1.59	0.115	0.51	0.611	**2.44**	**0.017**	1.22	0.222
Latitude	**2.53**	**0.013**	0.72	0.475	0.11	0.911	0.48	0.630
	*R* ^2^ = 0.2568 Adjusted *R* ^2^ = 0.2177		*R* ^2^ = 0.3423 Adjusted *R* ^2^ = 0.3077		*R* ^2^ = 0.2954 Adjusted *R* ^2^ = 0.2583		*R* ^2^ = 0.2586 Adjusted *R* ^2^ = 0.2196	

**FIGURE 2:**
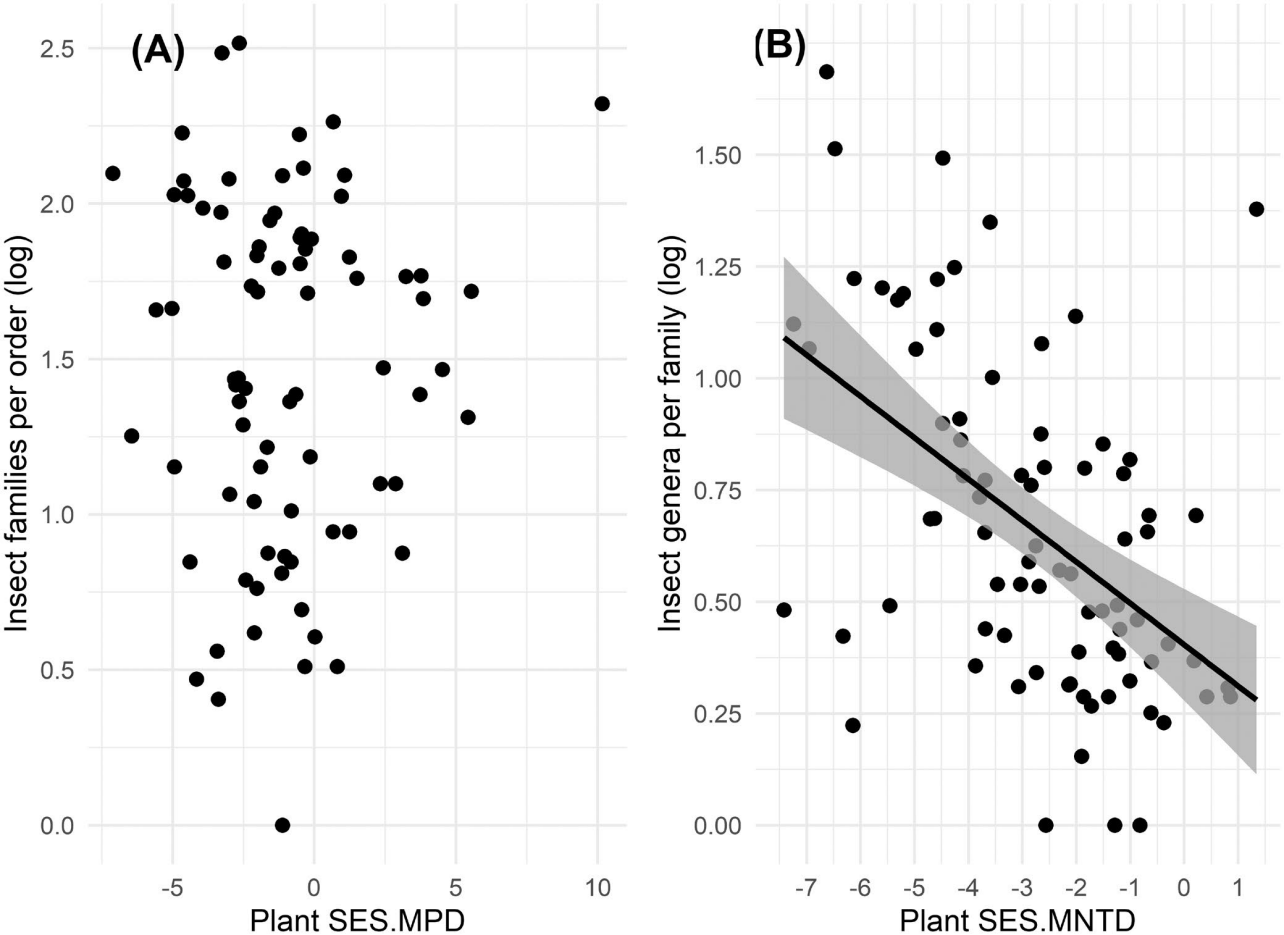
Correlations between (A) spatially detrended plant SES.MPD and the number of insect families per order, and (B) plant SES.MNTD and the number of insect genera per family across the studied islands (*n* = 81). Low values of plant indices indicate low PD, whereas low values of insect indices indicate high PD.

When considering the evolutionary relationships across the whole phylogeny, there is no detectable relationship between plant and insect diversity, as evidenced by the non‐significant correlation between plant SES.MPD and the number of insect families per order (correlation between spatially detrended variables; *R* = 0.08, *p* = 0.46, Figure 2A). However, high plant diversity in evolutionary more recent times (i.e., high SES.MNTD) is significantly associated with high recent insect diversity (i.e., low number of genera per family) (correlation between spatially detrended variables; *R* = −0.48, *p* < 0.001, Figure 2B).

**FIGURE 3:**
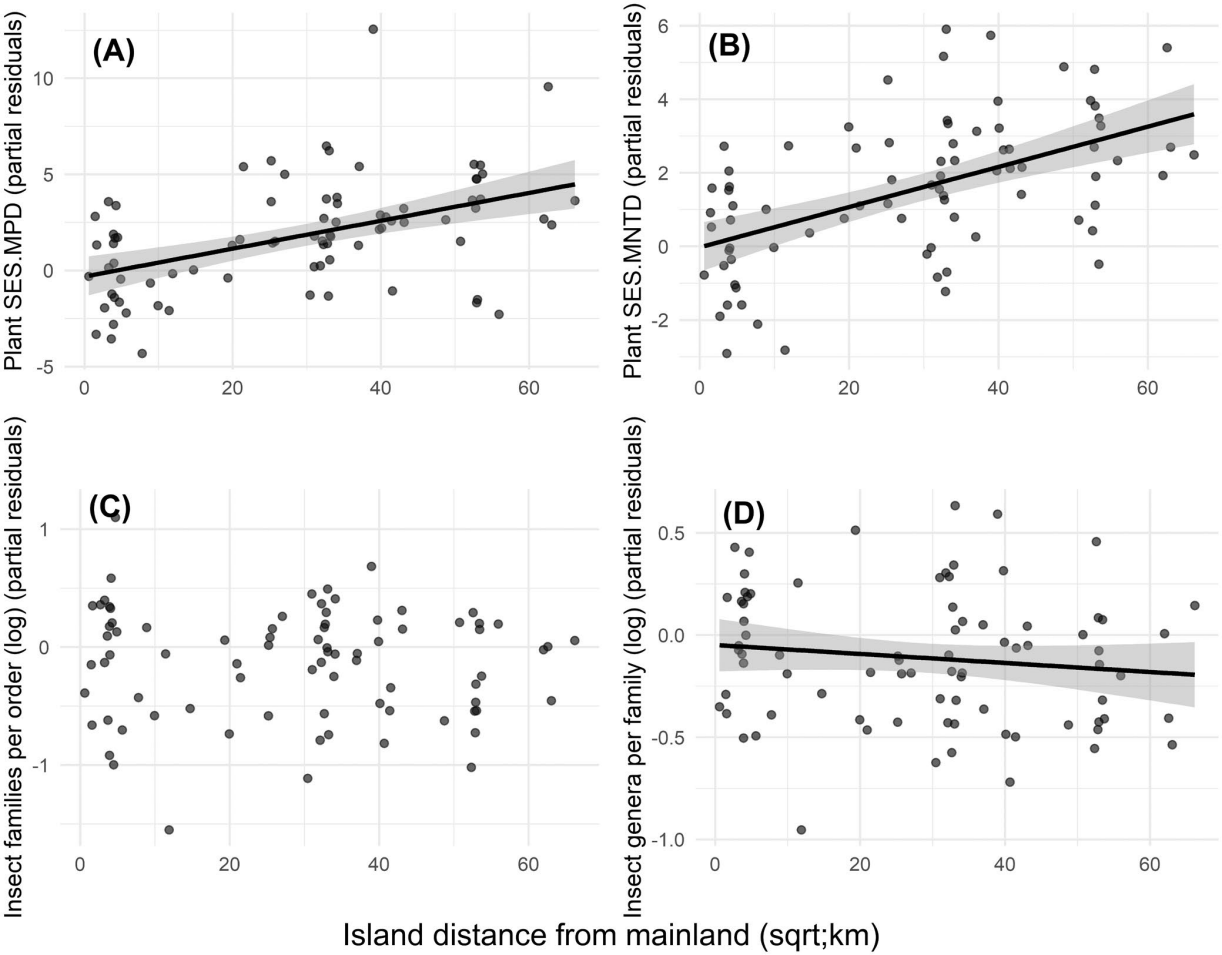
Linear regressions between the distance from mainland of the studied islands (*n* = 81) and the partial residuals of (A) plant SES.MPD, (B) plant SES.MNTD, (C) number of insect families per order, and (D) number of insect genera per family. Low values of plant indices indicate low PD, whereas low values of insect indices indicate high PD.

Both measures of plant diversity were significantly higher on the islands more distant from the mainland (plant SES.MPD: *t* = 3.02, *p* = 0.003, Figure 3A; plant SES.MNTD: *t* = 4.29, *p* < 0.001, Figure 3B). The number of insect families per order was not influenced by the distance of islands. Insect number of genera per family was marginally lower on distant islands, indicating more diverse insect communities (number of insect families per order: *t* = −1.53, *p* = 0.128, Figure 3C; number of insect genera per family: *t* = −1.85, *p* = 0.067, Figure 3D).

**FIGURE 4:**
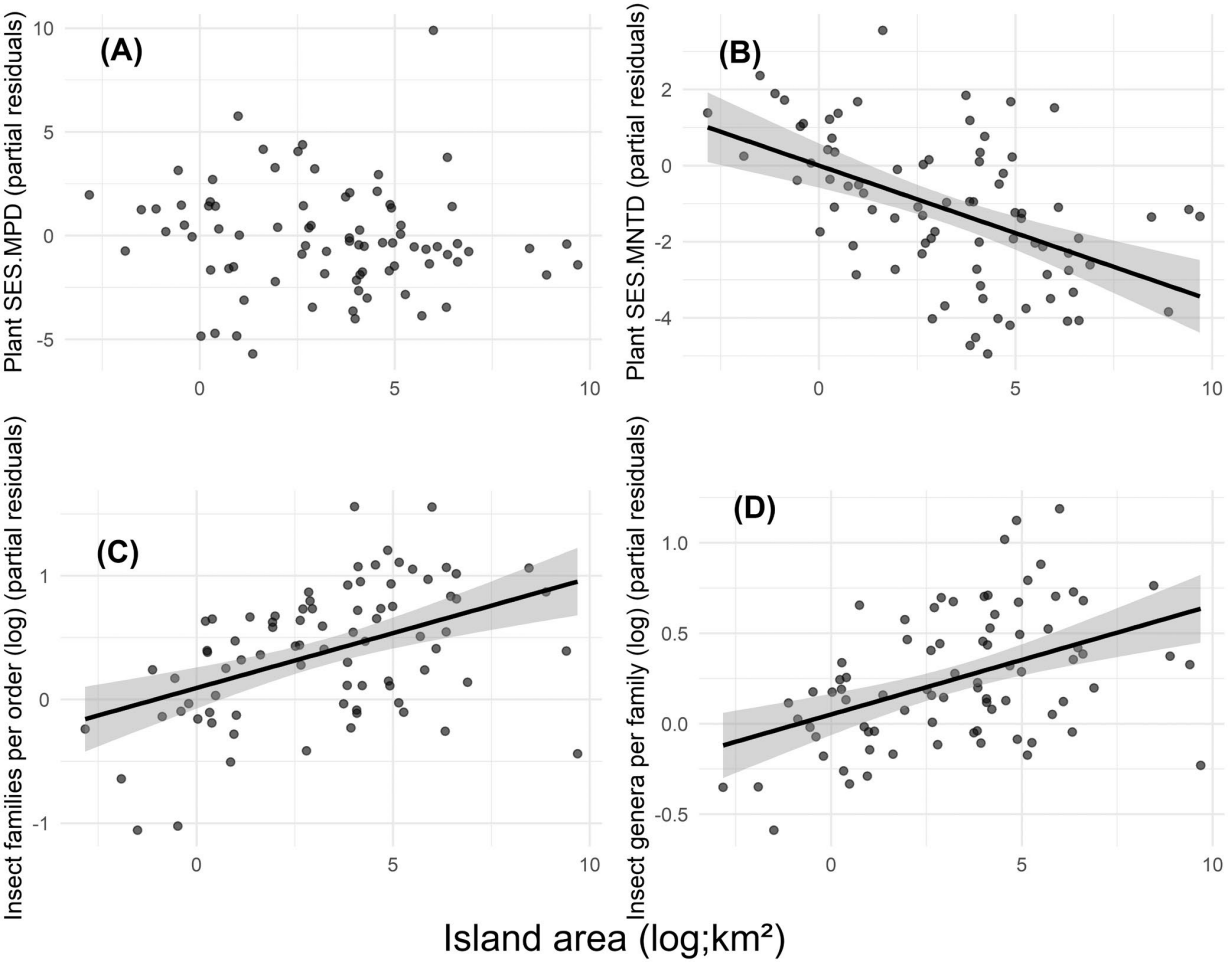
Linear regressions between the area of the studied islands (*n* = 81) and the partial residuals of (A) plant SES.MPD, (B) plant SES.MNTD, (C) number of insect families per order, (D) number of insect genera per family. Low values of plant indices indicate low PD, whereas low values of insect indices indicate high PD.

Plant and insect diversity was generally higher on small islands (plant SES.MNTD: *t* = −4.75, *p* < 0.001, Figure 4B; number of insect families per order: *t* = 6.67, *p* < 0.001, Figure 4C; number of insect genera per family: *t* = 5.11, *p* < 0.001, Figure 4D). However, there was no significant relationship between island area and plant SES.MPD (*t* = −0.51, *p* = 0.609, Figure 4A).

**FIGURE 5:**
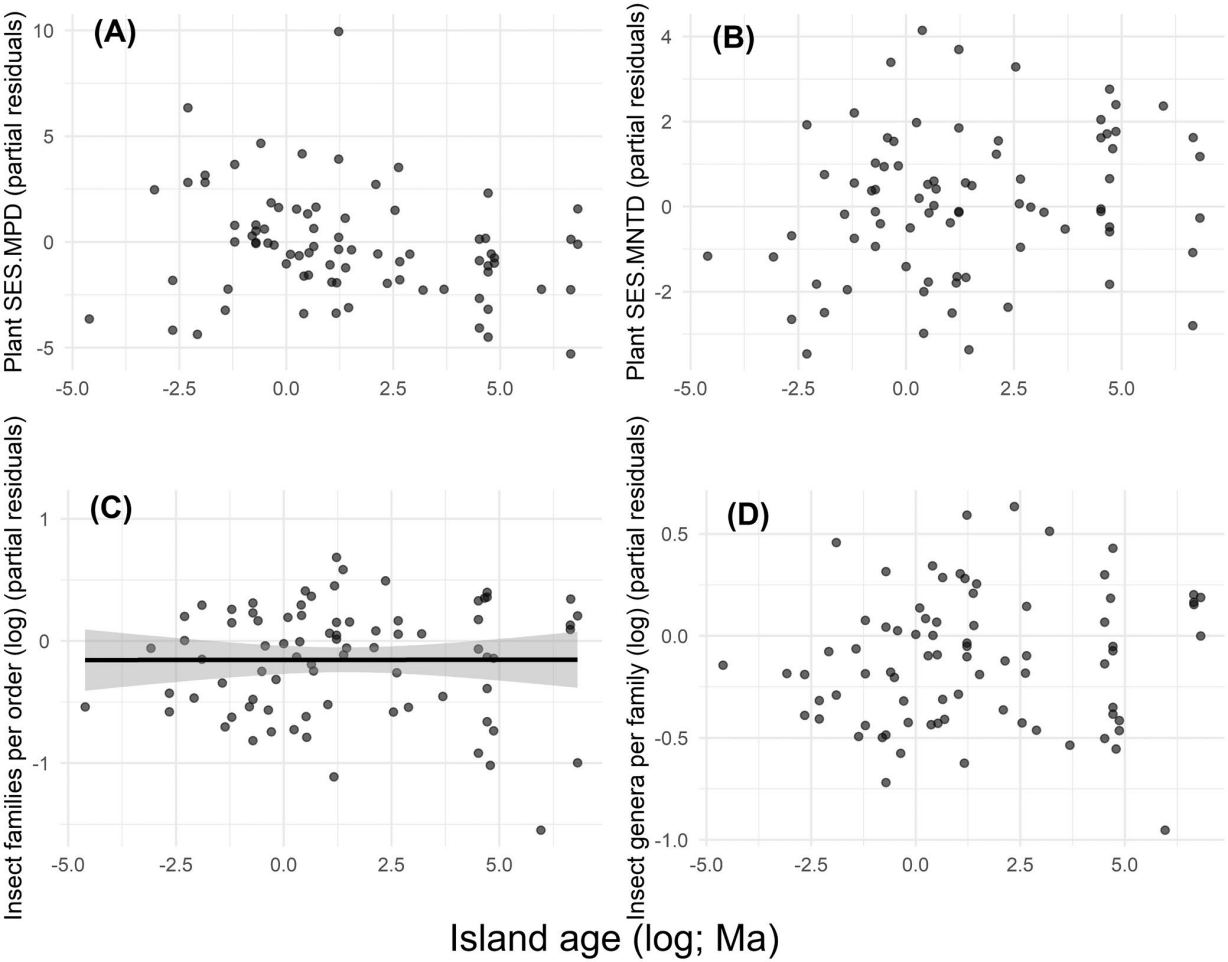
Linear regressions between the age of the studied islands (*n* = 81) and the partial residuals of (A) plant SES.MPD, (B) plant SES.MNTD, (C) number of insect families per order, (D) number of insect genera per family. Low values of plant indices indicate low PD, whereas low values of insect indices indicate high PD.

The number of insect families per order (*t* = 2.44, *p* = 0.017) was lower on young islands, indicating phylogenetically diverse insect communities (Figure 5C). However, we did not find significant relationships between island age and plant indices (plant SES.MPD: *t* = −1.59, *p* = 0.115, Figure 5A; plant SES.MNTD: *t* = 0.51, *p* = 0.611, Figure 5B) nor island age and the number of insect genera per family (*t* = 1.22, *p* = 0.222, Figure 5D).

**FIGURE 6:**
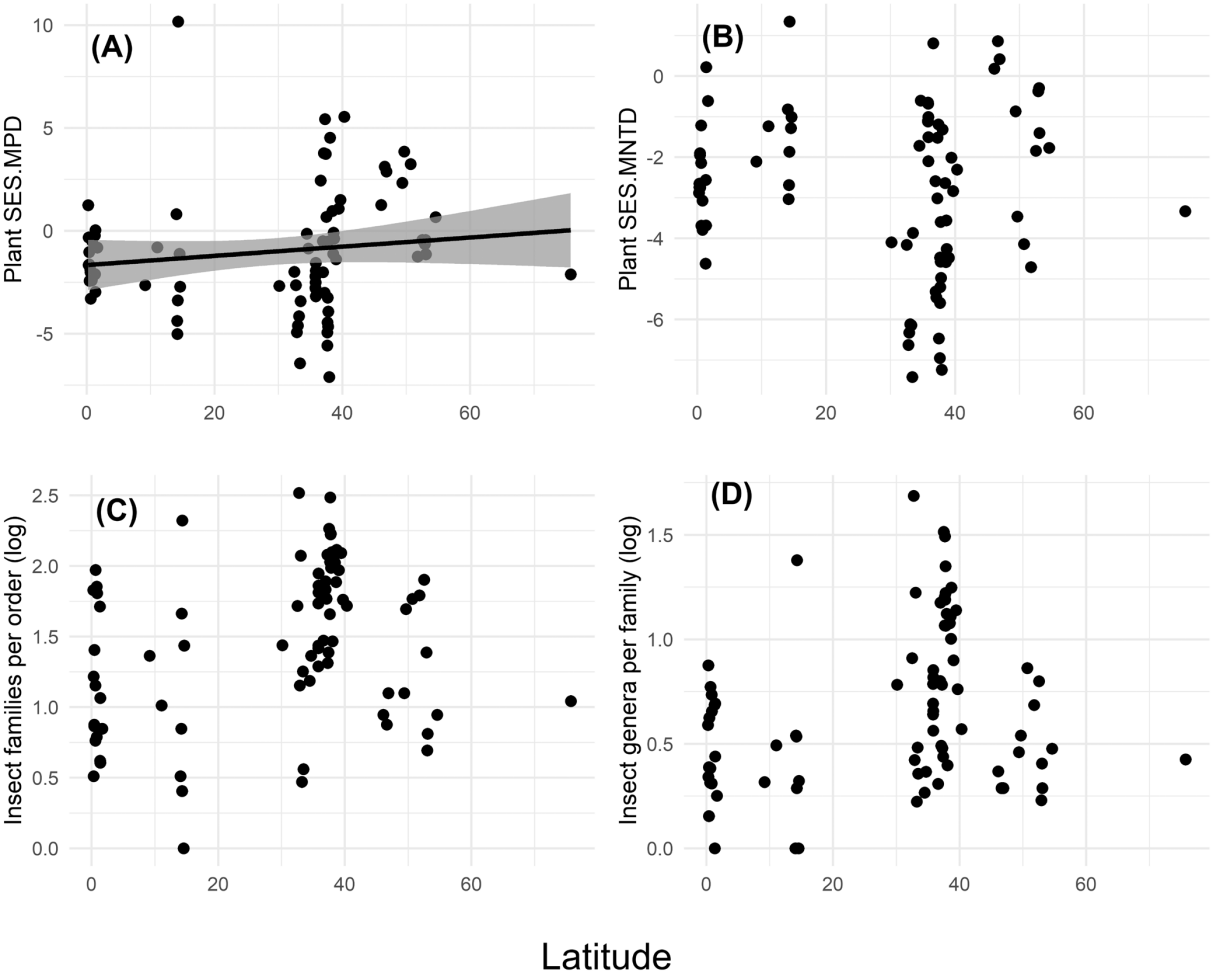
Linear regressions between the (absolute) latitudinal location of the studied islands (*n* = 81), and (A) plant SES.MPD, (B) plant SES.MNTD, (C) number of insect families per order, and (D) number of insect genera per family. Low values of plant indices indicate low PD, whereas low values of insect indices indicate high PD.

We did not find significant relationships between latitude and most plant and insect diversity metrics (plant SES.MNTD: *t* = 0.72, *p* = 0.475, Figure 6B; number of insect families per order: *t* = 0.11, *p* = 0.911, Figure 6C; number of insect genera per family: *t* = 0.48, *p* = 0.630; Figure 6D). However, plant SES.MPD was higher on high latitude islands (*t* = 2.53, *p* = 0.013, Figure 6A).

## 4 Discussion

We found that plant phylogenetic diversity in evolutionary recent times (i.e., high diversity among the tips of the phylogeny as reflected by SES.MNTD) is positively associated with the proxy measure of recent phylogenetic diversity in insects (i.e., low number of genera per family) on the studied islands, although the strength of this relationship depends on island features. In contrast, no detectable relationship emerges between the diversity of older lineages of plants and insects as shown by the absence of a correlation between plant SES.MPD and the number of insect families per order. These results indicate that diversification of plants and insects may have happened in parallel in evolutionarily recent times, after the rather independent arrival of ancient lineages of both groups on the islands. Alternatively, the relationship may have arisen from congeneric non‐native species of both groups colonizing the islands in evolutionary recent times. Indeed, a significant phylogenetic signal has been found worldwide in naturalized plant species, indicating that there are certain clades with an increased potential of their species to naturalize (Pyšek et al. 2017). These naturalizing plant clades may be associated with naturalizing insect clades.

We found that both measures of plant phylogenetic diversity (SES.MPD, SES. MNTD) increase with island isolation. High phylogenetic diversity of angiosperms has been previously recorded on remote islands (Weigelt et al. 2015) and explained by the assumption that well‐dispersing clades are widely spread across the phylogeny (Cavender‐Bares et al. 2009); thus, their arrival increases phylogenetic diversity. High phylogenetic diversity on distant islands has also been described in other taxa such as snakes (Portillo et al. 2019) and birds (Baiser et al. 2018). Our results show marginally higher insect diversity on distant islands when considering more recent diversifications, that is, lower number of genera per family, whereas the more ancient diversifications (number of families per order) are not influenced by island isolation. These findings imply that distant islands might host phylogenetically overdispersed communities of various taxa and at various levels of phylogeny.

There was generally high plant and insect phylogenetic diversity on small islands, except for plant SES.MPD which was not influenced by island size. High phylogenetic diversity on small islands may be explained by generally low species richness (MacArthur and Wilson 2001) which may not support high in situ speciation when compared to large islands. In situ speciation events add closely related species to the species already established (as described for mammals by Davies and Buckley (2011)) and, therefore, may lead to decreased phylogenetic diversity on large islands, though it needs to be tested in single clades. As we did not find a significant relationship between island size and plant SES.MPD (i.e., more ancient diversifications), we conclude that the high phylogenetic diversity of plants on large islands is caused by evolutionarily more recent radiations.

We found high phylogenetic diversity across more ancient lineages of insects, that is, low number of families per order, on young islands. This indicates that rather distantly related insect lineages arrive first and are able to establish on islands. It can be further suggested that in situ speciation events, potentially decreasing phylogenetic diversity, occur only on older islands as these are colonized for a longer period. Somewhat surprisingly, we could not detect significant relationships between island age and the measures of plant phylogenetic diversity. This indicates that plant lineages established on young islands are rather a random sample of lineages, possibly with a phylogenetic pattern in dispersal (Price and Wagner 2018), but not in establishment. The lack of a phylogenetic pattern in plants may also indicate that the mean age of the islands in our dataset could be too low compared to the ages of plant lineages, as shown for oceanic islands (Weigelt et al. 2015).

We could not find significant relationships between latitude and most metrics of plant and insect phylogenetic diversity, except for plant SES.MPD which was higher on high latitude islands. While some studies have found the latitudinal gradient in phylogenetic diversity in plants at a regional scale (e.g., Zu et al. 2023), no general gradient has been found at the global scale (Massante et al. 2019). Similar to our results, Massante et al. (2019) found high plant SES.MPD at high latitudes, however only in woody communities. In our dataset, the absence of the distantly related gymnosperms at < 32° N (Madeira) might be responsible for low SES.MPD at low latitudes.

We admit that attention must be given to the quality of insect data. As new species and genera are being described, previously identified taxa are susceptible to changed taxonomy. Therefore, insect species identified in this study might have changed taxonomy, leading to slightly different results. Furthermore, the potentially uncertain assessment of endemic genera on some remote islands can have an impact on the phylogenetic analysis.

Our results may have important implications for conservation purposes. First, we found that both plant and insect phylogenetic diversity are at least partly associated across the islands (plant SES.MNTD is correlated with the number of insect genera per family); therefore, it can be suggested that conserving plant phylogenetic diversity may be primordial to maintaining insect diversity. However, care must be taken to identify any non‐native species which are not subjected to conservation. Second, we found that small and distant islands generally harbor high phylogenetic diversity of both plants and insects. Consequently, we believe our results can help conservationists to maintain biodiversity by focusing the efforts on small and distant islands as these may be the hotspots of phylogenetic diversity. Although remote islands are harder to reach, their biodiversity is still influenced by human presence (e.g., tourism), invasive species, and climate change, leading their extraordinary unique biodiversity to collapse (Fernández‐Palacios et al. 2021) and the efforts to preserve biodiversity on such islands are challenging (Jupiter, Mangubhai, and Kingsford 2014).

## Author Contributions

**Thomas Leclère:** conceptualization (equal), data curation (lead), formal analysis (lead), investigation (equal), methodology (equal), validation (equal), visualization (equal), writing – original draft (lead). **Pille Gerhold:** conceptualization (equal), funding acquisition (lead), methodology (equal), project administration (lead), supervision (lead), writing – original draft (supporting), writing – review and editing (equal).

## Conflicts of Interest

The authors declare no conflicts of interest.

## Supporting information


Appendix S1.

Appendix S2.

## Data Availability

Data are available in Dryad: https://doi.org/10.5061/dryad.51c59zwdk.
