# Supplementary material for: Phylogenetic Diversity of Plant and Insect Communities on Islands
Source: Ecol Evol. 2024 Dec 2;14(12):e70660. doi: 10.1002/ece3.70660 (PMC11612024; doi:10.1002/ece3.70660)
Supplement: Supplementary file 2 — Appendix S2. [file ECE3-14-e70660-s001.docx]

# Evolutionary relationship between plants and insects: Insights from island communities

The data consists of 1 xlsv files.

The files can be opened using Excel.

"Supplementary_file_1" which contains 7 columns.

The first is the location of the island. The second is the latitude. The third is the scientific reference to establish the list of plant species for each location. The fourth column is the scientific name of the plant species (according to "The Plant List nomenclature"). The fifth column is the scientific reference to establish the list of insect species for each location. Finally, the sixth column is the list of insect species for each location. The seventh column is the scientific reference for the island age.

Abstract of the study:

Interactions between plants and insects have long fascinated scientists. While some plants rely on insects for pollination and seed dispersal, insects rely on plants for food or as a habitat. Despite extensive research investigating pair-wise species interactions, few studies have characterized plant and insect communities simultaneously, making it unclear if diverse plant communities are generally associated with diverse insect communities. This work aims to better understand the historical and evolutionary relationships between plant and insect phylogenetic diversity (PD) on islands. We hypothesized that phylogenetically diverse plant communities (i.e. high PD) support diverse insect communities, with the relationship varying with island isolation, area, age, and latitude. Species lists for plants and insects were compiled from the published literature, and plant PD was calculated using ´standardized mean pairwise distance´ (SES.MPD) and ´standardized mean nearest taxon distance´ (SES.MNTD). For insects, PD was estimated using the number of genera, families, and orders. We found that plant diversity in evolutionary recent times (SES.MNTD) is associated with recent insect diversity (number of genera), but no relationship was found between plant and insect diversity across whole phylogenies (plant SES.MPD vs. number of insect families). Distant islands generally support high PD of plants (high SES.MPD and SES.MNTD) and insects (low number of genera). Plant and insect PD was generally high on small islands, except for plant SES.MPD revealing no relationship with island size. Insect PD was somewhat higher on young islands (low number of families), whereas there was no relationship between island age and plant PD. Plant SES.MPD was higher on high latitude islands, yet we did not find significant relationships between the latitude and the metrics of insect PD, or plant SES.MNTD. These findings suggest that protecting high plant PD may also help conserve high insect PD, with a focus on small and distant islands as potential hotspots of phylogenetic diversity across multiple taxa.

## Description of the data and file structure

Same description as above.

## Sharing/Access information

Data was derived from the following sources:

\*

For species list:

Borges, P. A. V. (Ed.). (2008). Listagem dos fungos, flora e fauna terrestres dos arquiplagos da Madeira e Selvagens: = A list of the terrestrial fungi, flora and fauna of Madeira and Selvagens archipelagos. Secretario Regional do Ambiente e dos Recursos Naturais do Governo Regional da Madeira.

Borges, P. A. V., Cunha, R., Cristina Costa, A., & Gabriel, R. (2010). A List of the Terrestrial and Marine Biota From the Azores.

Broughton, D. A., & McAdam, J. H. (2005). A checklist of the native vascular flora of the Falkland Islands (Islas Malvinas): New information on the species present, their ecology, status and distribution 1. The Journal of the Torrey Botanical Society, 132(1), 115148. [https://doi.org/10.3159/1095-5674(2005)132\[115:ACOTNV\]2.0.CO;2](https://doi.org/10.3159/1095-5674\(2005\)132[115:ACOTNV]2.0.CO;2)

Cho, Y., Kim, Y., Lim, H.-M., Han, Y.-G., Choi, M.-J., & Nam, S.-H. (2011). A Faunistic Study of Insects of Uninhabited Islands in the Docho-myeon, Sinan-gun, Jeollanam-do, Korea. Korean Journal of Environment and Ecology, 25(5), 673684.

Chown, S. L., & Convey, P. (2016). Antarctic Entomology. Annual Review of Entomology, 61(1), 119137. [https://doi.org/10.1146/annurev-ento-010715-023537](https://doi.org/10.1146/annurev-ento-010715-023537)

Christensen, B. R., & Sutton, M. E. (2007). Ecology, management and research on Mokoia Island, Lake Rotorua, New Zealand: An abstract list and annotated bibliography. DOC Research & Development Series, 284, 93.

Court, D. J., Hardacre, A. K., & Lynch, P. A. (1973). The Vegetation Of The Aldermen Islands: A Reappraisal. Tane, 19, 4160.

Danks, H. V., & Byers, J. R. (1972). Insects and Arachnids Of Bathurst Island, Canadian Arctic Archipelago. The Canadian Entomologist, 104(1), 8188. [https://doi.org/10.4039/Ent10481-1](https://doi.org/10.4039/Ent10481-1)

Dreux, Ph. (1964). Observations sur la flore et la vgtation de llle aux Cochons (Archipel Crozet). Bulletin de la Socit Botanique de France, 111(78), 382386. [https://doi.org/10.1080/00378941.1964.10835487](https://doi.org/10.1080/00378941.1964.10835487)

Early, J. W. (1995). Insects of The Aldermen Islands. Tane, 35, 114.

Foreman, R. E. (1967). Observations on the Flora and Ecology of San Nicolas Island.

Fosberg, F. R. (1990). A review of the natural history of the Marshall Islands. Atoll Research Bulletin, 330, 1100. [https://doi.org/10.5479/si.00775630.330.1](https://doi.org/10.5479/si.00775630.330.1)

Frenot, Y., Gloaguen, J. C., Mass, L., & Lebouvier, M. (2001). Human activities, ecosystem disturbance and plant invasions in subantarctic Crozet, Kerguelen and Amsterdam Islands. Biological Conservation, 101(1), 3350. [https://doi.org/10.1016/S0006-3207(01)00052-0](https://doi.org/10.1016/S0006-3207\(01\)00052-0)

Godley, E. J. (1989). The flora of Antipodes Island. New Zealand Journal of Botany, 27(4), 531564. [https://doi.org/10.1080/0028825X.1989.10414138](https://doi.org/10.1080/0028825X.1989.10414138)

Greene, S. W., & Walton, D. W. H. (1975). An annotated check list of the sub-Antarctic and Antarctic vascular flora. Polar Record, 17(110), 473484. [https://doi.org/10.1017/S0032247400032459](https://doi.org/10.1017/S0032247400032459)

Hwang, J. H., Yim, M.-Y., An, S.-L., Paek, W.-K., & Lee, W.-H. (2022). Analysis of Seven Islands with Insect Fauna and Vascular Plant Flora in Gogunsan Archipelago, Korea. PROCEEDINGS OF THE NATIONAL INSTITUTE OF ECOLOGY OF THE REPUBLIC OF KOREA, 3(1), 2331. [https://doi.org/10.22920/PNIE.2022.3.1.23](https://doi.org/10.22920/PNIE.2022.3.1.23)

Johannesson, J. M. (1972). Insects of Red Mercury Island. Tane, 18, 6.

Johnson, P. N., & Campbell, D. J. (1975). Vascular Plants of the Auckland Islands. New Zealand Journal of Botany, 13(4), 665720. [https://doi.org/10.1080/0028825X.1975.10430354](https://doi.org/10.1080/0028825X.1975.10430354)

Jung, S.-Y., Park, S.-H., Nam, C.-H., Lee, H.-J., Lee, Y.-M., & Chang, K.-S. (2013). The Distribution of Vascular Plants in Ulleungdo and Nearby Island Regions (Gwaneumdo, Jukdo), Korea. Journal of Asia-Pacific Biodiversity, 6(1), 123156. [https://doi.org/10.7229/jkn.2013.6.1.123](https://doi.org/10.7229/jkn.2013.6.1.123)

Kami, K. S., & Miller, S. E. (1998). Samoan insects and related arthropods: Checklist and bibliography. Bishop Museum Press.

Lee, Y.-M., Park, S.-H., Jung, S.-Y., & Yang, J.-C. (2010). Study on the Vascular Plants Found in Nearby Island Regions of Ganghwado. Journal of Korean Nature, 3(2), 83101. [https://doi.org/10.1016/S1976-8648(14)60013-X](https://doi.org/10.1016/S1976-8648\(14\)60013-X)

Lim, J.-S., Park, S.-Y., Lim, J.-O., & Lee, B.-W. (2013). A Faunastic Study of Insects from Is. Ulleungdo and Its Nearby Islands in South Korea. Journal of Asia-Pacific Biodiversity, 6(1), 93121. [https://doi.org/10.7229/jkn.2013.6.1.093](https://doi.org/10.7229/jkn.2013.6.1.093)

Linsley, E. G., & Usinger, R. L. (1966). Insects of Galapagos. Proceedings of the California Academy of Sciences, 33(7).

Lososov, Z., marda, P., Chytr, M., Purschke, O., Pyek, P., Sdlo, J., Tich, L., & Winter, M. (2015). Phylogenetic structure of plant species pools reflects habitat age on the geological time scale. Journal of Vegetation Science, 26(6), 10801089. [https://doi.org/10.1111/jvs.12308](https://doi.org/10.1111/jvs.12308)

Lynch, P. A., & Ferguson, E. J. (1972). The Vegetation Of Red Mercury Island Part 1: The Plant Communities And A Vascular Plant Species List. Tane, 18, 9.

MacDonald, P. (1977). Insects From Moturoa Islands, May 1976. Tane, 23.

MAdam, J. H., & Brnnstrom, R. (2000). A Combined Checklist And Ecogeographic Conspectus For The Vascular Flora Of Saunders Island, Falkland (Malvinas) Islands. Anales Instituto Palagonia, 28, 5788.

Meads, M. J., & Fitzgerald, B. M. (2001). List of invertebrates on Mokoia Island, Lake Rotorua. Conservation Advisory Science Notes, 343, 11.

Meurk, C. D. (1975). Contributions to the Flora and Plant Ecology of Campbell Island. New Zealand Journal of Botany, 13(4), 721742. [https://doi.org/10.1080/0028825X.1975.10430355](https://doi.org/10.1080/0028825X.1975.10430355)

Miller, S. E. (1985). Entomology of The California Channel Islands. Proceedings of the First Symposium.

Moore, D. M. (1967). The Vascular Flora of the Falkland Islands. British Antarctic Survey Scientific Reports, 60.

Nam, C.-H., Park, S.-H., Jung, S.-Y., Oh, S. H., Hyun, J.-O., Kwon, H. J., & Chang, K. S. (2012). The Vascular Plants of Sinan-gun Jeollanam-do KoreaAphae-do (Is.), Bigeum-do (Is.) and Docho-do Island -. Journal of Korean Nature, 5(1), 6587. [https://doi.org/10.7229/jkn.2012.5.1.065](https://doi.org/10.7229/jkn.2012.5.1.065)

Park, S.-J., Kwon, H., Park, S.-K., Kim, D. S., & Park, D.-S. (2013). Comparative Insect Faunas between Ganghwado and Six Others Islands of West Coastal in Incheon, Korea. Journal of Asia-Pacific Biodiversity, 6(2), 197219. [https://doi.org/10.7229/jkn.2013.6.2.197](https://doi.org/10.7229/jkn.2013.6.2.197)

Park, S.-J., Song, I.-G., Park, S.-J., & Lim, D.-O. (2010). The Flora and Vegetation of Dokdo Island in Ulleung-gun, Gyeongsanbuk-do. Korean Journal of Environment and Ecology, 24(3), 264278.

Philbrick, R. N. (1972). The Plants of Santa Barbara Island, California. Madroo, 21(5), 329393.

Proche, ., Forest, F., Veldtman, R., Chown, S. L., Cowling, R. M., Johnson, S. D., Richardson, D. M., & Savolainen, V. (2009). Dissecting the plantinsect diversity relationship in the Cape. Molecular Phylogenetics and Evolution, 6.

Raven, P. (1963). A Flora of San Clemente Island, California. Aliso, 5(3), 289347. [https://doi.org/10.5642/aliso.19630503.08](https://doi.org/10.5642/aliso.19630503.08)

Rivire, F. (1979). La vie animale terrestre Takapoto. Journal de la Socit des ocanistes, 35(62), 1929. [https://doi.org/10.3406/jso.1979.2989](https://doi.org/10.3406/jso.1979.2989)

Robinson, B. L. (1902). Flora of the Galapagos Islands. Contributions from the Gray Herbarium of Harvard University, 24, 77269.

Ryu, J., Kim, Y.-K., Suh, S. J., & Choi, K. S. (2021). The Insect database in Dokdo, Korea: An updated version in 2020. Biodiversity Data Journal, 9, e62011. [https://doi.org/10.3897/BDJ.9.e62011](https://doi.org/10.3897/BDJ.9.e62011)

Sachet, M.-H. (1983). Takapoto Atoll, Tuamotu Archipelago: Terrestrial vegetation and flora. Atoll Research Bulletin, 277, 144. [https://doi.org/10.5479/si.00775630.277.1](https://doi.org/10.5479/si.00775630.277.1)

Sheard, J. W., & Geale, D. W. (1983). Vegetation studies at Polar Bear Pass, Bathurst Island, N.W.T. I. Classification of plant communities. Canadian Journal of Botany, 61(6), 16181636. [https://doi.org/10.1139/b83-174](https://doi.org/10.1139/b83-174)

Smith, R. I. L., & Prince, P. A. (1985). The natural history of Beauchne Island. Biological Journal of the Linnean Society, 24(3), 233283. [https://doi.org/10.1111/j.1095-8312.1985.tb00374.x](https://doi.org/10.1111/j.1095-8312.1985.tb00374.x)

Son, Dong Chan, Kim, Hyun-Jun, Lee, Dong-Hyuk, Jung, Su Young, Park, Su-Hyun, & Chang, Kae Sun. (2016). Flora of the Five West Sea Islands in Korea. Korean Journal of Plant Resources, 29(4), 434466. [https://doi.org/10.7732/KJPR.2016.29.4.434](https://doi.org/10.7732/KJPR.2016.29.4.434)

Sugerman, B. B. (1979). Additions to the List of Insects and Other Arthropods from Kwajalein Atoll (Marshall Islands). Proceedings, Hawaiian Entomological Society, 13(1), 5.

Tan, H. T. W. (1997). A Botanical Survey of Sungei Buloh Nature Park, Singapore. Gardens Bulletin Singapore, 49, 1535.

Thorne, R. (1967). A Flora of Santa Catalina Island, California. Aliso, 6(3), 177. [https://doi.org/10.5642/aliso.19670603.02](https://doi.org/10.5642/aliso.19670603.02)

Wace, N. M., & Dickson, J. H. (1965). The Terrestrial Botany of the Tristan da Cunha Islands. Philosophical Transactions of the Royal Society of London. Series B, Biological Sciences, 249(759), 273360.

Whistler, W. A. (1980). The Vegetation of Eastern Samoa. Allertonia, 2(2), 45157.

Whistler, W. A. (1983a). The Flora and Vegetation of Swains Island. Atoll Research Bulletin, 262, 125.

Whistler, W. A. (1983b). Vegetation and Flora of the Aleipata Islands, Western Samoa. Pacific Science, 37(3), 23.

Whistler, W. A. (1994). Botanical Inventory of the Proposed Tutuila and Ofu Units of the National Park of American Samoa (Technical Report No. 87).

Wright, A. E. (1977). Vegetation And Flora Of The Moturoa Island Group, Northland, New Zealand. Tane, 23, 20.

For islands age:

Abdel-Monem, A. A., Fernandez, L. A., & Boone, G. M. (1975). K-Ar ages from the eastern Azores group (Santa Mafia, Miguel and the Formigas Islands). Lithos, 8, 247254.

Bjrk, A. A., Bjrck, S., Cronholm, A., Haile, J., Ljung, K., & Porter, C. (2011). Possible Late Pleistocene volcanic activity on Nightingale Island, South Atlantic Ocean, based on geoelectrical resistivity measurements, sediment corings and 14 C dating. GFF, 133(34), 141147. [https://doi.org/10.1080/11035897.2011.618275](https://doi.org/10.1080/11035897.2011.618275)

Chevalier, J.-P., Denizot, M., Ricard, M., Salvat, B., Sournia, A., & Vasseur, P. (1979). Gomorphologie de latoll de Takapoto. Journal de la Socit des ocanistes, 35(62), 918. [https://doi.org/10.3406/jso.1979.2988](https://doi.org/10.3406/jso.1979.2988)

Chevallier, L., Rex, D. C., & Verwoerd, W. J. (1992). Geology and geochronology of Inaccessible Island, South Atlantic. Geological Magazine, 129(1), 116. [https://doi.org/10.1017/S0016756800008098](https://doi.org/10.1017/S0016756800008098)

Duncan, R., & Varne, R. (1988). The age and distribution of the igneous rocks of Macquarie Island. Papers and Proceedings of The Royal Society of Tasmania, 122(1), 4550. [https://doi.org/10.26749/rstpp.122.1.45](https://doi.org/10.26749/rstpp.122.1.45)

Fox, J. M., McPhie, J., Carey, R. J., Jourdan, F., & Miggins, D. P. (2021). Construction of an intraplate island volcano: The volcanic history of Heard Island. Bulletin of Volcanology, 83(5), 37. [https://doi.org/10.1007/s00445-021-01452-5](https://doi.org/10.1007/s00445-021-01452-5)

Geist, D. J., Snell, H., Snell, H., Goddard, C., & Kurz, M. D. (2014). A Paleogeographic Model of the Galpagos Islands and Biogeographical and Evolutionary Implications. In K. S. Harpp, E. Mittelstaedt, N. dOzouville, & D. W. Graham (Eds.), Geophysical Monograph Series (pp. 145166). John Wiley & Sons, Inc. [https://doi.org/10.1002/9781118852538.ch8](https://doi.org/10.1002/9781118852538.ch8)

Geldmacher, J., Hoernle, K., van den Bogaard, P., Zankl, G., & Garbe-Schnberg, D. (2001). Earlier history of the 70-Ma-old Canary hotspot based on the temporal and geochemical evolution of the Selvagen Archipelago and neighboring seamounts in the eastern North Atlantic. Journal of Volcanology and Geothermal Research, 111(14), 5587. [https://doi.org/10.1016/S0377-0273(01)00220-7](https://doi.org/10.1016/S0377-0273\(01\)00220-7)

Geldmacher, J., van den Bogaard, P., Hoernle, K., & Schmincke, H.-U. (2000). The 40 Ar/ 39 Ar age dating of the Madeira Archipelago and hotspot track (eastern North Atlantic): THE 40 Ar/ 39 Ar AGE DATING. Geochemistry, Geophysics, Geosystems, 1(2), n/a-n/a. [https://doi.org/10.1029/1999GC000018](https://doi.org/10.1029/1999GC000018)

Giret, A., Tourpin, S., Marc, S., Verdier, O., & Cottin, J.-Y. (2002). Volcanisme de lle aux Pingouins, archipel Crozet, tmoin de lhtrognit du manteau fertile au sud de locan Indien. Comptes Rendus Geosciences, 334, 481488.

Hayward, B. W., & Moore, P. R. (n.d.). Geology of the Aldermen Islands. Tane, 19.

Hayward, B. W., & Moore, P. R. (1972). Geology of Red Mercury Island (Whakau). Tane, 18.

Keating, B. H. (1992). The Geology of the Samoan Islands. In B. H. Keating & B. R. Bolton (Eds.), Geology and Offshore Mineral Resources of the Central Pacific Basin (Vol. 14, pp. 127178). Springer New York. [https://doi.org/10.1007/978-1-4612-2896-7_9](https://doi.org/10.1007/978-1-4612-2896-7_9)

Kim, S. W., Kwon, S., Ryu, I.-C., Jeong, Y.-J., Choi, S.-J., Kee, W.-S., Yi, K., Lee, Y. S., Kim, B. C., & Park, D. W. (2012). Characteristics of the Early Cretaceous Igneous Activity in the Korean Peninsula and Tectonic Implications. The Journal of Geology, 120(6), 625646. [https://doi.org/10.1086/667811](https://doi.org/10.1086/667811)

Kim, W. J., Oh, C. W., & Lee, S. H. (2021). The tectonic evolution of the Gogunsan Islands in the southwestern margin of the Gyeonggi Massif and its implication for the Neoproterozoic tectonic evolution relating to the Rodinia in the Northeast Asia. Lithos, 388389, 106054. [https://doi.org/10.1016/j.lithos.2021.106054](https://doi.org/10.1016/j.lithos.2021.106054)

Klgel, A. (2009). Klgel, Andreas (2009). Atlantic Region. In Rosemary Gillespie; David Clague (eds.). Encyclopedia of Islands. Berkeley: University of California Press. ISBN 978-0-520-25649-1.

Maund, J. G., Rex, D. C., Le Roex, A. P., & Reid, D. L. (1988). Volcanism on Gough Island: A revised stratigraphy. Geological Magazine, 125(2), 175181. [https://doi.org/10.1017/S0016756800009572](https://doi.org/10.1017/S0016756800009572)

McDougall, I., & Ollier, C. D. (1982). Potassium-argon ages from Tristan da Cunha, South Atlantic. Geological Magazine, 119(1), 8793. [https://doi.org/10.1017/S0016756800025681](https://doi.org/10.1017/S0016756800025681)

McDOUGALL, I., Verwoerd, W., & Chevallier, L. (2001). KAr geochronology of Marion Island, Southern Ocean. Geological Magazine, 138(1), 117. [https://doi.org/10.1017/S0016756801005039](https://doi.org/10.1017/S0016756801005039)

Nunn, P. D. (1998). Pacific Island Landscapes.

Oh, C. W., Kim, W. J., Lee, S. H., Lee, B. Y., Kim, J. S., & Choi, S. H. (2019). The Neoproterozoic and Cretaceous Tectonic Evolution and Important Geoheritages in the Gogunsan Archipelago. The Journal of the Petrological Society of Korea, 28(4), 251277. [https://doi.org/10.7854/JPSK.2019.28.4.251](https://doi.org/10.7854/JPSK.2019.28.4.251)

Quilty, P. (2007). Origin and evolution of the sub-Antarctic islands: The foundation. Papers and Proceedings of the Royal Society of Tasmania, 3558. [https://doi.org/10.26749/rstpp.141.1.35](https://doi.org/10.26749/rstpp.141.1.35)

Quilty, P., & Wheller, G. (2000). Heard Island and the McDonald Islands: A window into the Kerguelen Plateau. Papers and Proceedings of the Royal Society of Tasmania, 112. [https://doi.org/10.26749/rstpp.133.2.1](https://doi.org/10.26749/rstpp.133.2.1)

Scott, J. M., & Turnbull, I. M. (2019). Geology of New Zealands Sub-Antarctic Islands. New Zealand Journal of Geology and Geophysics, 62(3), 291317. [https://doi.org/10.1080/00288306.2019.1600557](https://doi.org/10.1080/00288306.2019.1600557)

Yong-Sun, S., Park, M.-E., & Park, K.-H. (2006). Ages and Evolutions of the Volcanic Rocks from Ulleung-do and Dok-do. The Journal of the Petrological Society of Korea, 15(2), 7280.

## Code/Software

Data analysis was conducted in R Studio (R Core Team 2022). Using mainly the following packages: "UTaxonstand", "V.PhyloMaker2" & "picante".
